# Cure as medicine’s constitutive aim: a defence of the refined curative thesis

**DOI:** 10.1007/s11019-026-10336-4

**Published:** 2026-03-11

**Authors:** Likhwa Ncube

**Affiliations:** Centre for Philosophy of Epidemiology, Medicine, and Public Health (CPEMPH), Faculty of Humanities, University of Johannesburg, 524, Auckland Park, 2006 South Africa

**Keywords:** Curative thesis, Inquiry thesis, Medical failure, Constitutive aim, Refined curative thesis, Philosophy of medicine

## Abstract

Medicine has persisted for thousands of years despite frequent therapeutic failures. What explains its persistence despite its failure to cure? I contend that the Inquiry thesis’s view – seeing medicine’s “core business” as prediction and understanding – rests on an unhelpful conceptual distinction between *main goal* and *core business*, along with an implausible “No Bullshit” premise. Instead, I support the refined Curative Thesis (RCT): medicine is united by a teleological (*constitutive*) aim to help the sick through socially organised and accountable interventions, even when cures are uncertain. Using conceptual analysis and analogies from comedy, mining, and policing, I demonstrate that professions can survive repeated failures in their main goals, revealing a symmetry: prediction and explanation often fail as well. This work is innovative in three ways: it discards “core business” as a defining tool, introduces a teleological boundary that accommodates epistemic diversity, and redefines prediction and understanding as instrumental goods intrinsic to medicine. It also relates RCT to other recent curative-focused and related accounts, such as Smart (Synthese 201(6):194, 2023); Varga (Science, medicine, and the aims of inquiry, Cambridge University Press, Cambridge, 2024); Stegenga (Care and cure, University of Chicago Press, Chicago, 2018), clarifying their significance.

## Introduction

Why has medicine persisted for millennia despite long periods of failure to reliably cure diseases? This question motivates Alex Broadbent’s “Puzzle of Ineffective Medicine” (PIM) (Broadbent 2019a, p. 56). Historically, medical traditions have often failed to produce consistent therapeutic results (Porter 1997; Wootton 2006). Indeed, significant curative success is largely a 20th-century phenomenon (Marcum 2011; Stegenga 2018b). Yet medicine has remained a distinct and respected institution (Broadbent 2019a).

Broadbent’s conclusion is subtle but important: he does not deny that curing is the *goal* of medicine. Rather, he denies that curing is also its *core business*. The Curative Thesis, as he frames it, treats these as identical, claiming that both the goal and the core business of medicine are “to heal the sick—to cure” (2019a, p. 35). Broadbent rejects this identity and instead proposes that the core business lies in two competencies: understanding and prediction. These, he argues, explain medicine’s persistence despite a historically poor record of cures.

His reasoning draws on the “Argument from the Persistence of Ineffective Medicine” (APIM), which can be summarised as follows:

1. If curing were the core business of medicine, long-term failure to cure would have undermined medicine’s persistence.
2. Medicine has persisted despite prolonged and widespread therapeutic failure.
3. Therefore, curing cannot be the core business of medicine (2019a, pp. 59–60).

The first premise rests on what Broadbent calls the ‘No Bullshit premise’: that a profession’s *core business* must be something it reliably accomplishes. Broadbent writes, “The No Bullshit Premise says that, if the core business of medicine were cure, then medicine would not persist if it were ineffective at cure” (2019a, p. 77). It is this premise that my paper directly challenges.

My argument against the ‘No Bullshit premise’ is twofold. Firstly, I oppose the framing that underpins both the Curative thesis and the Inquiry thesis, especially the distinction between the main goal and the core business of medicine. I believe the idea of a core business is conceptually superfluous and unhelpful. Secondly, I reject the ‘No Bullshit premise’ as simply wrong. It is factually inaccurate, even assuming, for discussion’s sake, that the distinction between the main goal and core business holds. It isn’t accurate to claim that professions that fail to consistently perform their core business (whatever that may be) should eventually disappear. I illustrate this through comparisons with comedy, mining, and policing. Ultimately, I highlight that Broadbent claims prediction and understanding define medicine, but I contend that medicine’s identity lies in its ongoing focus on cure. Even when curing isn’t reliably achievable, the aim of restoring or improving health continues to guide medical practice and research, shaping what medicine fundamentally is. I package and defend this view as the refined Curative Thesis (RCT).

The RCT builds on and distinguishes itself from other recent defences of the Curative Thesis (Smart 2023) and from other engagements with the Curative Thesis in the recent literature (Fuller 2022; Stegenga 2018a; Varga 2024). The novelty here is threefold. First, I use cross-professional analogies (comedy, mining, and policing) to challenge the core assumption of Broadbent’s APIM, showing that persistence despite frequent failure is not unique to medicine and does not require redefining its nature. Second, while others have engaged with Broadbent’s distinction between main goal and core business (Harris 2018; Metz, 2018; Ncube 2025; Smart 2023), none have recognised that, conceptually, it is better to set aside. Third, I articulate a refined, teleology-based Curative Thesis that preserves the centrality of cure while accommodating epistemic diversity. Methodologically, this paper uses conceptual analysis and philosophical argumentation. It does not include an empirical study of medical practices or a detailed ethnographic comparison of different medical traditions. Its goal is to clarify the conceptual basis for defining medicine.

The paper is organised as follows. Section 2 presents the different perspectives on the nature of medicine according to the Curative and Inquiry Theses. Section 3 evaluates the APIM by analogy to other professions. Section 4 unambiguously rejects Broadbent’s framing that distinguishes between the *main goal* and *core business* of medicine, claiming the distinction is conceptually unhelpful. Section 5 introduces the refined Curative Thesis. Section 6 redefines the roles of prediction and understanding as secondary to cure. Section 7 explains the difference between competence and legitimacy. Section 8 offers a new interpretation of medicine’s continued existence, emphasising its enduring commitment to cure. Section 9 wraps up the discussion.

## Competing accounts of the nature of medicine

At the heart of the recent debate about the nature of medicine, two contrasting views have emerged prominently: the Curative Thesis and the Inquiry Thesis (Broadbent 2018a, b; Fuller 2021; Harris 2018; Metz, 2018; Metz and Harris 2018; Smart 2023; Varga 2024). More recently, Varga (2023, 2024) has introduced an autonomy-based account into the debate (see Sect. 5, below). Broadbent’s framing of the disagreement between the Curative and Inquiry theses highlights critical differences, not just semantic but also philosophical. It questions how we perceive medicine’s goals, the skills that support its main activities, and the social legitimacy that has maintained medicine as a strong profession for millennia. Each thesis provides a unique conceptual basis for understanding what defines *medicine*, particularly in situations where curative results are uncertain or unavailable.

The Curative Thesis asserts that medicine is “the sustained and organized effort to heal the sick or prevent them getting sick in the first place” (Broadbent 2019a, p. 35). Importantly, the version Broadbent critiques equates the *goal* of medicine with its core business, both as to heal and cure the sick. Broadbent himself describes this perspective as: “Both are to heal the sick—to cure. Hence the name” (2019a, p. 35). He provides a broad definition of cure:

I am happy to count as a cure any intervention that is reasonably effective at alleviating the ill effects of a disease, incapacity, reduced life span, and suffering. On this very generous definition, even painkillers count as cures. (Broadbent 2018b, p. 291)

After setting the Curative thesis in the above manner, Broadbent proceeds and accepts that cure is medicine’s *goal*. What he rejects is that cure is also its *core business*. This is the crux of his disagreement with the Curative Thesis: while cure may be the desired end-state, the central defining activity of medicine (its core business), in his view, lies elsewhere.

The Inquiry Thesis is Broadbent’s proposed alternative. It claims that medicine’s defining feature is its consistent demonstration of two core competencies:

1. Understanding: the capacity to meaningfully explain health and illness,
2. Prediction: the capacity to make accurate, often conditional, prognoses about health states.

According to Broadbent, these competencies – rather than curative successes – link various medical traditions across different eras and cultures, from Western biomedicine to African Traditional Medicine (ATM) and others in between, to all be recognisable as medicine. In this perspective, medicine’s competence is not based on curing but on its ability to diagnose and prognosticate effectively. Broadbent arrives at the Inquiry Thesis using the Argument from the Persistence of Ineffective Medicine (APIM), which is summarised as follows:

1. If curing were the core business of medicine, long-term failure to cure would have undermined medicine’s persistence.
2. Medicine has persisted despite prolonged and widespread therapeutic failure.
3. Therefore, curing cannot be the core business of medicine (2019a, pp. 59–60).

The first premise rests on what Broadbent calls the ‘No Bullshit premise’: the idea that a profession’s core business must be something it reliably accomplishes, otherwise it wouldn’t persist (2019a, p. 60). This assumption is central to his challenge of the Curative thesis and is precisely the crux of my argument against the Inquiry thesis. For clarity, I distinguish three terms that will be used throughout:

- [Main] Goal – the ultimate intended end of a practice.
- Core business – the defining activity through which a profession’s social identity is constituted, what medicine *actually does*.
- Constitutive Aim – is the normative goal that defines the institution and unifies its practices across different contexts, such as medicine’s focus on cure, rather than any fixed “core business”.

With these distinctions in place, the dispute between the Curative Thesis and the Inquiry Thesis comes into sharper relief. Broadbent accepts cure as a *goal* but rejects it as the *core business*. I reject the claim that such a distinction (main goal and core business) is conceptually illuminating and helpful^1^. Moreover, even if we grant that the distinction holds, I reject the idea that frequent failures to cure (as medicine’s core business) undermine that status. Using analogies from other professions (in the next section), I show that persistence without consistent success is neither anomalous nor incompatible with a practice’s defining aim. Thus, I challenge Broadbent’s ‘No Bullshit premise’ directly.

## Assessing the APIM through analogical reasoning

Broadbent’s argument, from the Persistence of Ineffective Medicine (APIM), relies on a claim that may seem straightforward at first but is ultimately philosophically weak: a profession cannot persist if it consistently fails in its core business. He states this clearly:

There is no easy response to be had by asserting that the core business of medicine is trying to heal the sick. Normally, one’s core business is something one actually does, not something one merely tries to do. Blacksmiths do more than try to make horseshoes, and taxi drivers do more than try to take you places. (Broadbent 2019a, p. 34)

This reasoning is based on what he calls the ‘No Bullshit premise’: the idea that a profession’s core business must be something it *reliably* accomplishes (2019a, p. 60). His comparison to taxi drivers is intended to be straightforward: if most taxi drivers failed to deliver passengers to their destinations, the industry would collapse. Similarly, if medicine’s core business were curing, its frequent failures to do so should have led to its collapse.

Nevertheless, this analogy masks key differences in how professional persistence functions. Taxi driving involves short-term, transactional tasks, in which repeated failures can erode trust between driver and passenger. Conversely, medicine is a long-term, socially embedded institution centred on moral and aspirational aims. Its legitimacy depends less on ensuring specific results and more on sustaining an organised, credible effort toward achieving a valued goal. Accepting, for the sake of argument, Broadbent’s strategy of appealing to analogy to support the ‘No bullshit premise’, likewise, let us examine our own examples.

Think about comedy. The core business of a comedian could be thought of as making people laugh. However, even the best comedians often fail – jokes flop, audiences lose interest, and performances can become awkwardly silent. Despite these setbacks, comedy as a profession remains and thrives. Comedians keep their careers, attract audiences, and play a significant cultural role, even when they don’t always succeed in making people laugh. This shows that a profession can be defined more by its *aspirational* goal than by perfect results. The commitment to try humour persists, and audiences continue to support comedy because they appreciate the effort, even when success isn’t guaranteed.

Mining also provides a useful comparison. The profession exists to extract valuable resources from the ground. However, many mining efforts result in no commercially viable deposits; oil-drilling projects often fail; entire operations can be unsuccessful. Despite this, mining companies continue to operate, attract investments, and are regulated by governments. This persistence is normal, driven by the value placed on resource extraction and the understanding that failure is part of the process. As in medicine, the importance of the goal sustains the institution despite risks and setbacks.

Policing serves as a third analogy, that is, to preserve public order, enforce laws, and reduce crime. Despite ongoing issues with crime in some areas and the police often not meeting their goals, the institution is rarely abandoned. Instead, it undergoes reforms, expansions, and defences. The enduring presence of policing is rooted in its normative focus on justice and order. Even when it fails, its existence is justified by the continued importance of these objectives. This illustrates how the profession is judged not only by its success but also by its sustained commitment to its main goals.

Indeed, these analogies differ from medicine in morally significant ways, Broadbent might retort. Medicine directly addresses human suffering and life-or-death stakes, whereas comedy, mining, and even policing operate in less urgent domains. Yet this difference strengthens, rather than weakens, the argument underpinning the analogy. The higher the moral importance of an aim, the more reason societies have to sustain the institution despite inconsistent success. Suppose we are willing to maintain comedy or mining despite repeated failure. In that case, we have even more substantial grounds to keep medicine, whose aim is partly to preserve and restore life. Moreover, it shouldn’t take a careful look to see that Broadbent’s possible rejoinder is disingenuous. His examples of taxi driving and blacksmithing could also be argued to differ from medicine in morally significant ways.

Looking again at analogies, what do they suggest about the nature of medicine? As indicated, these analogies challenge the ‘No Bullshit premise’ fundamental to the APIM. Professions can endure despite often failing to meet their defining aims. They persist because their goals are socially valued, institutionally supported, and culturally anchored. Medicine should be viewed through this lens. The fact that medical treatments sometimes do not cure does not mean that curing is not their main goal. On the contrary, medicine’s structure – its training, institutions, research priorities, and public ethos – remains centred on the goal of curing (Pellegrino and Thomasma 1981, 1993; Tosam 2014). Similar to comedy, mining, and policing, as shown earlier, the importance of its goal explains why it persists, the seriousness of its commitments, and the institutional frameworks that uphold it (Zola 1972).

This section argues that Broadbent’s taxi analogy is flawed because it mischaracterises the conditions for persistence. In the next section, I provide additional detail on why medicine might persist despite high failure rates, thus directly further challenging the ‘No Bullshit premise’. I also demonstrate that Broadbent’s distinction between the main goal and the core business of medicine is unhelpful and should be abandoned.

## Rejecting the distinction between ‘Main Aim’ and ‘Core Business’

Broadbent’s distinction between the aim of medicine and its core business, where “core business” is understood as *what medicine actually does*, seems to be less effective than it appears. More precisely, it is somewhat conceptually redundant and does not significantly aid understanding of the connections that link medicine across historical periods and medical traditions. The reason is straightforward: “What medicine actually does” can vary significantly over time, yet medicine remains recognisable. If so, then “core business” cannot be the basis of medicine’s identity. Instead, this foundational role is played by constitutive aims, primarily curative aims.

Let us begin with two stress tests. First, imagine advanced AI tools radically changing medicine: diagnostic reasoning becomes automated, treatment is dynamically personalised, triage is handled by learning systems, bedside procedures are reorganised around decision-support pipelines, and surgery might become largely robotic. Such a transformation *could make* the core activities, procedures, workflows, and epistemic methods of medicine appear very different from those of earlier eras. Second, consider alien medicine systems that are completely unfamiliar to ours. Their clinicians may not use familiar imaging, pharmacology, or surgery; their disease concepts might not align with ours; and their interventions could be unrecognisable compared to current biomedicine.

Observe how these scenarios challenge Broadbent’s idea of a “core business”. Without a clear core linking our medicine with AI or alien medicine, attempts to define one tend to be either too narrow (excluding future possibilities) or too broad (merely restating a goal). For example, if we define the core business as “applying biomedical science,” alien medicine might not be biomedical at all, and our own medicine could shift from “applied science” to what looks more like “engineered control” under AI. Alternatively, if we say it’s “diagnosis and treatment,” we’re already talking about aims, since these are *medical* activities only in relation to their purpose.

Despite the wide variability in activity, all these practices still qualify as medicine. This is the key point. Their unity doesn’t stem from sharing stable tasks, tools, or routines, but from being organised around a fundamental goal that makes their diverse practices intelligible as parts of the same type of institution. The only way to understand this unity is through their *constitutive aims* – specifically, curative aims. When viewed appropriately, curative aims define medicine as the socially and institutionally oriented practice of intervention to improve the health of the sick, even when success isn’t guaranteed. The crucial insight is that the notion of a fixed core activity (Broadbent’s core business) has become obsolete. As what is actually done becomes historically and technologically adaptable, it no longer effectively defines the institution.

A parallel point becomes clearer if we change the domain of discussion. To emphasise this, consider the institution of a ‘corporation’. It is very plausible, and almost cliché, to state that a corporation has a main goal, such as generating profit for shareholders (setting aside complicated stakeholder models). But then ask: what is a corporation’s *core business?* The answer is not straightforward. Some corporations produce cars, others run social media platforms, some manage logistics, others sell insurance, and some conduct biotech research. This question is nonsense because corporations encompass a wide range of core activities, and knowing these specifics is not necessary to understand the institution’s fundamental aim. We can identify the type of institution by its constitutive aim and normative framework, even if its actual activities differ greatly. The same principle applies to medicine. If medicine is an institution unified by a constitutive aim, then its “core business” is at best secondary, or even misleading, leading us to search for a core activity profile that does not exist.

This is why I distance myself further from Broadbent by criticising the significance of this distinction. “Core business” either merges into “aim” (once abstract enough to pass the AI/alien test) or becomes an unstable sociological label (once specified enough to seem informative). In either case, it does not serve any important conceptual purpose. It fails to anchor identity through change, establish principled boundaries, or clarify why certain practices are considered medical rather than merely health-related (precise terminology) (Fagerberg 2023). The constitutive aim handles that; core business does not.

Varga (2024) highlights an insightful point related to the constitutive aim. The NATO case is particularly illustrative because it demonstrates the inadequacy of focusing solely on an institution’s “core business” as Broadbent defines it, which refers to a fixed set of activities NATO “actually does”. These activities often vary over time due to changes in history, geopolitics, and technology. A more effective way to understand NATO’s identity is to consider its *purpose*: the constitutive aim that unifies and explains why its evolving activities remain connected to a single institution. This reflects the core argumentative approach: when activities change, the aims remain consistent with the institution’s unity (Varga 2024, pp. 86–87).

Varga primarily uses the concept of constitutive aim to distinguish it from aim, highlighting that institutions may have many aims but only a few constitutive ones. As Varga explains: “But while it might be true that complex activities and institutions tend to have a plurality of aims, it is nevertheless possible for them to have a single or at least very few constitutive aims” (Varga 2024, p. 86). I extend this idea by using constitutive aims not just to categorise aims but to challenge the need for a separate “core business” category altogether. If constitutive aims account for institutional identity across different practices, then “core business” is not an essential fact about the institution but a contingent description of how that aim is enacted in a particular context. Therefore, “core business” is conceptually unnecessary for the definitional purpose.

Finally, contrary to the “No Bullshit premise”, it’s important to clarify why medicine persists despite high failure rates or limited ability to cure. Several factors sustain medicine socially, even if its curative capacity is modest: hope among patients and families (Ncube 2025); the significant risk asymmetry that makes “doing something” more psychologically and politically appealing than doing nothing (Misselbrook 2024); monopolistic licensing that protects medical professions from market exit (Abbott 1988); the tendency to interpret placebo effects, natural disease progression, or regression to the mean as signs of effectiveness, especially for conditions like the flu that tend to improve spontaneously (Hróbjartsson and Gøtzsche 2001); human biases and selective memory, favouring successes over failures (Nickerson 1998); the authority of white coats, institutions, and credentials (Rehman et al. 2005); and medicine’s tangible benefits such as care, reassurance, triage, palliation, and coordinated treatment, even when it doesn’t cure (Cassel 1982). In summary, medicine isn’t “bullshit,” but assuming that persistence indicates strong curative success is too hasty. Institutions persist because they are socially vital, normatively supported, and psychologically stabilising, even if their primary outcomes remain uncertain.

## The refined curative thesis

Having earlier dismantled the “No Bullshit premise” premise and subsequently dismissed the distinction between the main aim and core business as unhelpful, we can now turn to supporting the positive thesis. In this section, I articulate a defence of a version of the Curative Thesis, which I refer to as the refined Curative Thesis (RCT). The goal is to address the shortcomings of both the naive Curative Thesis and Broadbent’s Inquiry Thesis.

The RCT can be stated as follows:

*Medicine is the socially organised*,* institutionally legitimised*,* and morally structured effort to restore*,* maintain*,* or improve human health through interventions directed at beneficial change*,* even when such interventions do not reliably or consistently result in cure.*

A key innovation of the RCT is its emphasis on *aims* rather than *outcomes*. The naive Curative Thesis might be seen as defining medicine solely by its results: medicine is about curing, so only successful cures qualify as genuine medicine. This view is too restrictive and can be challenged by historical examples (Porter 1997; Wootton 2006). The RCT shifts the definition to a teleological perspective: a practice is considered “medical” if it aims to improve health through intervention, regardless of whether the goal is always achieved. This shared orientation explains why both technologically advanced hospitals and remote rural clinics, surgical wards, and palliative care units are united: they all share a structured commitment to achieving beneficial health outcomes.

By focusing on intention and orientation rather than specific epistemic or technical criteria (core business), the RCT allows various medical traditions to coexist without diluting the concept. For instance, in ATM, healing might focus on restoring harmony using herbal remedies and spiritual practices (Ngubane 1977). These methods might not always be based on physiological or pathological mechanisms, especially from a systems perspective (Broadbent and Ncube 2023), but they are organised around the goal of improving health (James et al. 2018; World Health Organization, 2013). Within the RCT framework, ATM is acknowledged as medical without needing to fulfil biomedical epistemic standards like prediction or understanding (Ncube 2025).

This sets the RCT apart from Broadbent’s Inquiry Thesis, which may exclude certain traditions lacking its preferred prediction and understanding forms. It also differentiates the RCT from radical pluralism, which might include non-medical practices due to the absence of clear teleological or institutional boundaries. A common concern with teleological accounts is that they can become trivial if medicine is defined solely by its purpose; then, anything could be considered medicine. The RCT addresses this issue by insisting on three specific conditions.

1. Health-directed aim – The activity should focus on enhancing or preserving health. Actions done only for profit, political power, or entertainment do not meet this criterion.
2. Social organisation and institutional accountability – The practice must be embedded within a recognised and regulated structure, whether formal (licensing bodies) or informal (community-sanctioned healer roles).
3. Intervention for beneficial change – The practice must involve some form of *health-related* action, even if indirect or non-invasive. Placebo effects, symbolic comfort, or passive observation count only when integrated into a broader therapeutic project.

These conditions clarify why Western biomedicine and ATM are regarded as medicine, whereas astrology, fortune-telling, or purely symbolic rituals are not. The RCT also clarifies the classification of borderline activities, such as epidemiological modelling or genetic mapping. Although these are scientific pursuits involving disease-related knowledge, they qualify as “medical” only when integrated into projects focused on health improvement (Boorse 2016). For example, a geneticist working alone on mapping the human genome is not practising medicine. Still, if the same geneticist collaborates within a clinical team to guide treatment, the difference is quite clear.

Having articulated and defended the RCT, it’s worth noting the inspiration behind this thesis. Although the RCT is a new perspective, it builds on previous accounts that, to varying degrees, have recognised and positioned cure at the core of medicine. The RCT fits within a family of intervention and goal-focused accounts that resist defining medicine solely by its epistemic abilities. Instead of viewing prediction and understanding as the nature of medicine, these related perspectives (which inspired the RCT) emphasise that medicine is a practical endeavour: it centres on its *purpose* – improving health by addressing sickness through interventions – and also maintains principled boundaries to prevent medicine from expanding into health-related activities outside its scope.

Smart’s (2023) Best Available Intervention Thesis (BAIT) aligns closely with the view that medicine is primarily about intervention rather than merely acquiring knowledge. The phrase “best available” emphasises that medicine involves choosing the most suitable intervention from multiple options amid uncertainty, guided by normative standards. The RCT supports this perspective and aligns with Smart’s thesis.^2^ Suggesting that medicine aims for the “best available intervention” introduces a quality standard that isn’t inherently obvious: best *compared to what goal*? Smart hints at this but doesn’t fully develop the idea that “best available intervention” makes sense only within a teleological framework where medicine’s purpose is healing. The RCT underscores this: ranking interventions as ‘better’ depends on the goal of cure or healing, which bestows “best” with normative significance. Without this purpose, “best available” could become just a procedural term based on guidelines or evidence, rather than a concept grounded in medicine’s *main goal*.

Elsewhere, Varga’s (2023, 2024) autonomy-based account can also be viewed as supportive of the RCT, as it complicates Broadbent’s clear two-option framework of Inquiry versus Curative theses. Varga’s approach is not merely about knowledge nor limited to a narrow view of cure as biological repair. It emphasises that ‘making the sick better’ often involves restoring or safeguarding agency, self-governance, and the ability to pursue a valued life. This perspective is ethically valuable for the RCT: it clarifies why many areas of clinical practice – such as rehabilitation, psychiatry, pain management, and palliative care – are considered medical even if they don’t directly address the causal basis of a disease. However, focusing on autonomy alone can risk expanding medicine too far (since many non-medical institutions also promote autonomy). The RCT thus considers autonomy a vital internal good and guiding principle in healing, but maintains a clear boundary: medicine’s goals must be achieved through socially organised, accountable interventions aimed at health. This institutional boundary prevents the view from dissolving into a vague pluralism where any autonomy-enhancing activity is labelled “medicine.”

Stegenga (2018a) is perhaps the most direct predecessor to the RCT’s teleological strategy. The RCT is inspired by Stegenga’s explicit appeal to medicine’s aims – “care and cure” – and by the way those aims are linked to a hybrid account of disease and intervention (2015). Stegenga’s argument that medicine aims to ‘care and cure’ is also teleological, grounded in his hybridism account of disease. He explicitly claims that medicine aims to improve health by targeting disease. He writes, “At least some of these interventions are not properly ‘medical’, since they are not targeting genuine diseases with the aim of improving a person’s health” (Stegenga 2015, p. 34). The basic idea is that, since a condition must have both a naturalistic and a normative element to be a disease, an intervention must target at least one of those elements to be effective (hence, ‘cure’ when targeting the natural basis of disease and ‘care’ when targeting the harm basis). His book *Care and Cure: An Introduction to Philosophy of Medicine* (2018a) has his supposed aim of medicine built into the very title.

This teleological framing supports the RCT’s core insistence that medicine is defined by its orientation – improving health by acting on disease – while allowing plurality in *how* that aim is pursued (cure and care-directed strategies). The RCT also draws on Stegenga’s broader scepticism about intervention effectiveness: if confidence in many interventions ought to be tempered, then medicine cannot be defined by guaranteed success (Stegenga 2018b). The RCT therefore emphasises curative striving rather than outcome dependence: medicine can remain medicine even when it fails, so long as it acts within accountable institutions towards healing-directed ends.

Smart (2023) emphasises intervention, Varga (2024) highlights the ethical aspects of “getting better,” and Stegenga (2018a) focuses on aims such as care and cure. The RCT builds on these perspectives by explicitly stating the teleological goal and incorporating principled institutional boundaries that uphold medicine’s moral identity, without narrowing its scope solely to biological cures. Lastly, we’ll briefly consider some potential objections to my presentation and defence of the RCT, along with my responses.

Objection **1**: The RCT may legitimise ineffective practices. I respond that the RCT isn’t a blank cheque for any health-related practice. Legitimacy depends on institutional structures that regulate and assess interventions, allowing for critique and reform when efficacy is absent. Objection **2** suggests that “Beneficial change” in RCT is too vague. I explain that the concept is intentionally adaptable to fit various medical contexts. What’s beneficial for a terminal cancer patient isn’t the same as for a child with an infection. This flexibility reflects real-world medical practice without losing conceptual clarity. Lastly, Objection **3** claims that the RCT understates epistemic competence by replacing Broadbent’s dual emphasis on prediction and understanding as core business in medicine. I argue that the RCT maintains epistemic competence as a vital tool but does not give it absolute priority. Prediction and understanding are important because they help achieve healing goals. A practice can be valid in medicine without meeting Western epistemic standards, but it can’t be valid without aiming for beneficial health outcomes.

The following section draws on this objection **3**. Further, it clarifies the instrumental roles that prediction and understanding play in the refined curative thesis (RCT), rather than the definitional roles assumed by the Inquiry thesis.

## Prediction and understanding as instruments of the curative aim

Broadbent’s Inquiry thesis emphasises prediction and understanding as central to medicine. For instance, he writes “The inquiry thesis accepts that the goal of medicine is cure, but asserts that its core business is understanding” (Broadbent 2019b, p. E106). It draws in part on history, noting that medicine has often succeeded in diagnosis and prognosis despite the lack of effective cures. Therefore, Broadbent contends that these capacities better explain medicine’s endurance than cures do (2019a, pp. 65–66). The RCT recognises the significance of prediction and understanding but does not regard them as the defining features of medicine. They are vital tools, but their importance lies in their role in achieving the ultimate goal of curing. Just as manual dexterity is vital to painting but not its defining trait, prediction and understanding are essential to medicine but do not constitute its core.

Let us begin with understanding. In medicine, understanding can range from mechanistic knowledge, such as cellular pathology, to phenomenological insights, such as patient experience (Carel 2016). It helps practitioners identify causes, develop interventions, and explain outcomes. However, in medical practice, understanding is rarely pursued purely for its own sake. Its value is usually judged by how well it guides treatment, enhances outcomes, or informs prevention strategies (Fuller 2022; Stegenga 2018a). Even if an explanatory model doesn’t suggest a therapeutic pathway, it may still be valuable in biology or philosophy of science, but it doesn’t count as a medical breakthrough. The RCT frames understanding as instrumental – it’s useful because it supports or improves health-related interventions.

Next, let’s examine prediction. It functions similarly, with accurate forecasts supporting triage, resource distribution, and patient choices. Nonetheless, prediction alone, without therapeutic relevance, doesn’t constitute a medical achievement. For example, a climate scientist predicting an increase in malaria cases due to rising temperatures isn’t practising medicine; their prediction becomes “medical” only when used to inform interventions – such as mosquito control or vaccination efforts. Broadbent’s “necrotic finger” example illustrates this well (2019a, pp. 67–68). The doctor’s prediction of tissue death is considered a medical act because it occurs within a care setting: it involves explanation, patient management, and decision-making about palliative or preventive actions. Without such integration into a therapeutic framework, prediction remains detached.

Why does RCT’s instrumental framing matter? It clarifies why patients value physicians for their predictive and interpretive skills. Patients seek relief from these abilities, not merely knowledge or foresight. Prognosis and explanations are appreciated because they provide guidance, reassurance, or a pathway to intervention (Marcum 2011). Without this link, epistemic skills lose much of their moral and professional importance in medicine. This framing also explains why similar skills in non-medical fields – like actuarial risk modelling or historical epidemiology – don’t carry the same medical status. The key difference is *teleology*: in medicine, understanding and prediction are linked to a socially sanctioned effort aimed at improving health.

A key strength of the RCT, compared with the Inquiry Thesis, is its straightforward assignment of prediction and understanding roles, thereby avoiding overextension. While Broadbent may be right that these skills are valuable and often more common than cures, their frequency does not make them defining. Many professional skills, like record-keeping, communication, and administration, are widespread without being central to identity. Furthermore, understanding and prediction are not necessarily more consistently present than cures. For instance, in AI, many effective treatments (“cures” by Broadbent’s broad definition) exist, yet understanding remains limited. Conversely, for diseases with no effective interventions, prediction and understanding are also limited. A doctor may have perfect prediction and understanding, but if these do not lead to action or improvement, both doctor and patient might see the encounter as a failure. This failure relates more to the goal (curing) than to knowledge itself, making the RCT better at capturing the emotional and moral complexities of medicine.

Prediction and understanding are essential tools in medicine, but they do not uncover its true nature. Their legitimacy stems from their role in supporting the ethical and practical goal of healing. In the next section, I will discuss how recognising this distinction between *competence* and *legitimacy* further enhances the RCT and clarifies the limits of medical practice and research.

## Competence and legitimacy in medicine

A key strength of the RCT is its ability to clarify a frequently blurred distinction in philosophy of medicine: that between medical competence and professional legitimacy (Miller et al. 2000; Miller and Brody 1995).^3^ For example, Broadbent’s Inquiry Thesis tends to conflate these concepts, assuming that recognition of competence – especially when no cure is available – is enough to define what medicine is. The RCT clarifies that although competence and legitimacy are connected, they should be analysed as separate.

Let’s analyse Broadbent’s reasoning linking competence to the definition of medicine. He highlights that patients and colleagues may still see a practitioner as competent even if a cure isn’t achieved. His example of a ‘necrotic finger’ demonstrates this: the doctor foresees tissue death, explains the process, but doesn’t provide treatment. Broadbent argues that the patient’s ongoing view of this as “medical” supports his Inquiry Thesis, which posits that medicine’s primary role is epistemic engagement rather than the production of therapeutic effects. He notes, “What makes this a competent medical opinion? My proposal is that it is the fact that the doctor understands what is going on. She can explain what is happening to the finger” (Broadbent 2019a, p. 67). The RCT, however, challenges this conclusion. Competence is a local, context-specific assessment of skill in a specific task.

In contrast, legitimacy is a broader, structural and normative standing that is consistent with the ethical objectives of the institution and profession. A doctor’s skill in making a prognosis does not imply that prognostication forms the essence of medicine. Similarly, just as a general lauded for a strategic retreat is valued within the scope of a larger mission, in medicine, the overarching mission remains the goal of curing.

Within the RCT framework, epistemic skills like diagnosis and prognosis are valued when they support a curative approach. The ‘necrotic finger doctor’ is deemed competent not only for making accurate *prediction*s but also because these predictions form part of a care strategy, such as symptom management, infection prevention, or preparing patients for subsequent stages. This principle also applies in palliative care: a physician who cannot cure but relieves pain, aids in decision-making, and preserves dignity is regarded as competent *because* these actions align with the overall goal of enhancing health or quality of life.

Again, this distinction clarifies why some highly skilled individuals are not classified as medical professionals. Epidemiologists, data scientists, and actuaries can accurately forecast health outcomes, but outside direct health interventions, they do not practise medicine (Boorse 2016). Conversely, a healer can be deemed legitimate without extensive scientific knowledge, as seen in some forms of ATM. For example, a *Sangoma*^4^ who communicates with ancestors may not use biomedical explanations but remains respected in their community because their role is culturally and institutionally focused on healing (Cumes 2013). The RCT accommodates this diversity without diluting the core definition: legitimacy depends on participation in a recognised, socially endorsed healing process.

In RCT, legitimacy is upheld by institutional frameworks such as licensing boards, regulations, customs, and ethical standards that ensure that medical practices serve moral goals. These structures protect public trust by verifying practitioners’ competence and dedication to healing. Even a talented autodidact with diagnostic skills cannot practise medicine without official approval. This emphasises medicine as a collectively regulated moral pursuit within the community, rather than merely a set of individual abilities. It also impacts global health policy.

Finally, understanding the difference between competence and legitimacy is crucial for defining medicine. Broadbent’s Inquiry Thesis risks defining it solely by competencies that can often be performed outside the medical field. In contrast, the RCT emphasises these skills but roots legitimacy in the profession’s teleological purpose. This shows how medicine can evolve, adopt new knowledge, and withstand failures without losing its core identity. Its stability depends not on the universal applicability of prediction and understanding but on how these are integrated into the moral and institutionally legitimised goal of healing. The following section revisits Broadbent’s PIM within this framework, illustrating that a teleological focus on cure, rather than on epistemic universals, best explains medicine’s resilience and unity.

## Reframing the puzzle: cure as teleological anchor

We can now revisit Broadbent’s PIM with a deeper understanding of how the curative goal functions within medicine. As Broadbent highlights, the puzzle stems from a historical reality: medicine has often failed to provide reliable cures, yet it has endured across different eras and cultures. He interprets this to mean that curing is not the core business of medicine (a notion I have shown to be unhelpful), and that its emphasis on prediction and understanding better explains its continued existence. He argues, “My task, then, is to show that inquiry can be explained in a way that shows that medicine has been much better at this than it has been at cure” (Broadbent 2019a, p. 65). The RCT offers a different perspective: medicine endures not because it consistently achieves cures, but because it is teleologically oriented toward the *goal* of curing. The profession’s resilience is attributed to the moral importance and social value attached to that goal, which are independent of immediate success rates.

Broadbent’s argument that historical failure signals a change in definition relies on an output-focused view of professional identity. If the core output isn’t achieved, the profession must have a different defining characteristic. In contrast, the RCT I support proposes a goal-centred (teleological) approach: a profession’s nature is determined by its *purpose*, not solely by what it consistently accomplishes. Many critical social practices function this way. For instance, law seeks justice despite occasional miscarriages; education aims at learning despite inconsistent results. Similarly, medicine strives for healing, even when cures are only partial or infrequent. Failures reflect issues with the curative goal, not that the goal is unimportant.

The Inquiry Thesis argues that prediction and understanding account for persistence because they are always present, even when cures are not available. However, a more plausible way to think of it is as follows: persistence in medicine is better understood as stemming from society’s inability to abandon the pursuit of health. When biomedicine fails, people turn to alternative medical traditions – not because they seek explanation or prediction, but because they want relief from illness and suffering (Harris 2018). The desire to heal is universal: it crosses cultures and eras. It drives public funding, supports professional training, and underpins practitioners’ moral authority (Beauchamp 2001). This is why medicine endures in many different forms, with varying epistemic bases but unified by its focus on curing.

Crucially, RCT also redefines what failure means in medicine. According to the Inquiry Thesis, failure happens when predictions or understanding are flawed or incomplete. However, in the RCT framework, failure can occur even when there is solid epistemic competence – if it doesn’t result in health improvements. For example, a doctor might accurately predict disease progression and fully understand its pathology. Still, if they cannot intervene effectively, both the doctor and the patient could regard the situation as a failure. The moral and emotional weight of that failure makes sense only if medicine is understood to aim at healing, not merely epistemic achievement.

Finally, the teleological approach of the RCT accommodates epistemic diversity without falling into the trap of “anything goes” pluralism. Traditions such as Ayurvedic medicine and Western biomedicine can both be regarded as medicine within the RCT framework, as they are institutionally centred on healing, despite differences in their explanatory models and methods. Meanwhile, purely symbolic or exploitative practices can be rejected if they lack a genuine health-oriented purpose or institutional legitimacy. Therefore, reframing the PIM through the RCT addresses this issue without redefining medicine in purely epistemic terms. Persistence despite the fallibility of cures is not unusual; it is the natural pattern of a practice tied to a valued goal that societies refuse to abandon. While prediction and understanding are important, they serve as tools to support that goal, not as the core definition of medicine.

## Conclusion

This paper defended the refined Curative Thesis (RCT) against Broadbent’s Inquiry Thesis by reframing the Puzzle from Ineffective Medicine (PIM) as a question about aims rather than outputs. While Broadbent correctly highlights the often absent clear cures in medicine and emphasises prediction and understanding, he mistakenly concludes that these epistemic skills are the core business of medicine. The RCT offers a more philosophically satisfying and normatively persuasive perspective, claiming that cure is a goal. It rejects the naive idea that cure must also be part of what Broadbent calls the ‘core business’ of medicine, and that legitimacy depends only on consistent success. Instead, the RCT explains why medicine persists despite fallibility, grounding its identity in its *teleological purpose*: the ongoing, socially recognised, and morally vital goal of healing.

## Footnotes

1 I am grateful to an anonymous reviewer for recommending that I avoid overreliance on Broadbent’s framing, which distinguishes the main goal from the core business. In my view and that of the reviewer, this distinction is not very illuminating conceptually and, in the worst case, is seriously mistaken.

2 While in agreement with Smart’s curative slant, this work diverges from Smart’s in rejecting the conceptually limited framing of Broadbent on both the Curative and Inquiry theses, which requires distinguishing between ‘main goal’ and ‘core business’ of medicine. This work differs from Smart’s precisely on this point, as Smart, even in the title of his paper, *The core business of medicine: a defence of the best available intervention* (2023), remains bound by Broadbent’s conceptually unhelpful framing.

3 In contrast to Pellegrino (2001), Miller et al. (2000) and Miller & Brody (1995) argue that medicine’s complexity and its legitimate scope cannot be reduced to a single essential goal, such as healing or health promotion. Moreover, their reasoning blurs the distinction between medical competence and professional legitimacy. See also Boorse (2016), who makes the same point.

4 In African Traditional Medicine, particularly in southern Africa, a *Sangoma* is a healer or diviner who diagnoses illness, usually through ancestral or spiritual consultation, and provides treatment or guidance through rituals and herbal medicines.
